# The Surreptitious Burden of Nonalcoholic Fatty Liver Disease in the Elderly in the Asia-Pacific Region: An Insight from the Global Burden of Disease Study 2019

**DOI:** 10.3390/jcm12206456

**Published:** 2023-10-11

**Authors:** Pojsakorn Danpanichkul, Siwanart Kongarin, Sarunpakorn Permpatdechakul, Natchaya Polpichai, Kwanjit Duangsonk, Worapaka Manosroi, Nathorn Chaiyakunapruk, Omar Y. Mousa, Donghee Kim, Vincent L. Chen, Karn Wijarnpreecha

**Affiliations:** 1 Immunology Unit, Department of Microbiology, Faculty of Medicine, Chiang Mai University, Chiang Mai 50200, Thailand; 2 Faculty of Medicine, Chiang Mai University, Chiang Mai 50200, Thailand; 3 Department of Internal Medicine, Weiss Memorial Hospital, Chicago, IL 60640, USA; natchayap.md@gmail.com; 4 Department of Microbiology, Faculty of Medicine, Chiang Mai University, Chiang Mai 50200, Thailand; 5 Division of Endocrinology, Department of Internal Medicine, Chiang Mai University, Chiang Mai 50200, Thailand; 6 Clinical Epidemiology and Clinical Statistics Center, Faculty of Medicine, Chiang Mai University, Chiang Mai 50200, Thailand; 7 Department of Pharmacotherapy, College of Pharmacy, University of Utah, Salt Lake City, UT 84112, USA; nathorn.chaiyakunapruk@utah.edu; 8 IDEAS Center, Veterans Affairs Salt Lake City Healthcare System, Salt Lake City, UT 84108, USA; 9 Division of Gastroenterology and Hepatology, Department of Internal Medicine, Mayo Clinic, Rochester, Mayo Clinic Health System, Rochester, MN 55902, USA; 10 Division of Gastroenterology and Hepatology, Stanford University School of Medicine, Stanford, CA 94305, USA; dhkimmd@stanford.edu; 11 Division of Gastroenterology and Hepatology, Department of Internal Medicine, University of Michigan, Ann Arbor, MI 41809, USA; 12 Division of Gastroenterology and Hepatology, Department of Medicine, University of Arizona College of Medicine, Phoenix, AZ 85004, USA; 13 Division of Gastroenterology and Hepatology, Department of Internal Medicine, Banner University Medical Center, Phoenix, AZ 85006, USA

**Keywords:** steatotic liver disease, elderly, Asia, epidemiology, geriatrics

## Abstract

Nonalcoholic fatty liver disease (NAFLD) represents a significant health threat worldwide. The aging population and a rise in metabolic syndrome in Asia might influence the epidemiology of NAFLD among the elderly. However, there is a lack of understanding of the burden and recommendations for NAFLD in this group. Our study sought to investigate the trends in the NAFLD burden among the elderly in the Asia-Pacific region. We employed data from the Global Burden of Disease 2019 study for an in-depth analysis of the prevalence and disability-adjusted life years (DALYs) along with age-standardized rate (ASR) associated with NAFLD in elderly populations (age 65–89 years) across the Asia-Pacific region, including the Southeast Asia (SEA) and Western Pacific (WP) regions, from 2010 to 2019. This study also examined the trends and disparities in NAFLD burden across different nations and sexes. In 2019, there were over 120 million cases of NAFLD in the elderly in the Asia-Pacific region. The ASR of prevalence was higher in SEA compared to WP (36,995.37 vs. 32,821.78 per 100,000). ASR of prevalence increased with annual percentage change (APC) +0.95% in the WP while it increased by +0.87% in SEA. During the study period, the ASR of DALYs decreased in SEA (APC −0.41%) but remained stable in the WP region. The burden of NAFLD in the elderly population in Asia-Pacific has increased, underscoring the timely intervention to tackle this high and rising burden.

## 1. Introduction

Nonalcoholic fatty liver disease (NAFLD) has been considered a public health concern globally [1,2]. It significantly contributes to the burden of liver cirrhosis, hepatocellular carcinoma, and liver transplantation necessities, subsequently imposing a substantial financial burden on the healthcare system [3]. The principal risk factors for NAFLD are lifestyle-associated factors, including diabetes and obesity. In addition, with aging, individuals with NAFLD undergo age-associated deteriorative shifts, such as mitochondrial dysfunction and telomere shortening [4]. In terms of clinical outcomes, it is noteworthy that the elderly cohort exhibits poorer outcomes than their younger counterparts, including higher degrees of fibrosis, steatohepatitis, and cancer [5,6]. The unique characteristics of NAFLD in older adults, combined with the rising incidence of metabolic syndrome and an aging population, contribute to a considerable health challenge linked to NAFLD [7].

Asia, home to over half of the global population, is projected to have the largest elderly demographic compared to the West [8]. Furthermore, the aging of people in Asia is occurring faster than that in the West, posing challenges to the healthcare system. In addition, the unabated burden of viral hepatitis persists, which exerts a synergistic role in liver injury [9]. Despite the growing health challenges tied to NAFLD, there has not been a report on its implications for the elderly in the Asia-Pacific region. We conducted a study to determine temporal trends in prevalence and disability-adjusted life years (DALYs) due to NAFLD in older adults in the Asia-Pacific region from 2010 to 2019.

## 2. Materials and Methods

### 2.1. Data Source

This research utilized the data from the Global Burden of Disease Study (GBD) 2019, a comprehensive effort to gauge the burden caused by 369 diseases and 87 risk factors across 204 countries/territories [10]. Data on the counts and age-standardized rates (ASRs) of NAFLD prevalence and disability-adjusted life years (DALYs) in individuals aged 65–89 between 2010 and 2019, categorized by sex, age, country, and region, were extracted from a database called the GlobalHealth Data Exchange query tool. The GHDx tool is an online data collaborative effort involving multiple countries and overseen by the Institute for Health Metrics and Evaluation to facilitate ongoing research on global health data.

### 2.2. Estimation Methods

NAFLD in older individuals refers to those between the ages of 65–89 and is identified using the ICD-10 code as K 76.0 or designated in the GBD database as “total burden of NAFLD”. Even though the name has changed after the multi-society Delphi consensus, from NAFLD to metabolic dysfunction associated steatotic liver disease (MASLD), the data from the NAFLD period can still be used because there’s little difference between the two terms [11,12]. The primary objective of this study is to evaluate the impact of NAFLD on the elderly population in the Asia-Pacific region. Specifically, our focus extends to the Southeast Asia (SEA) and Western Pacific (WP) territories, as categorized by the World Health Organization (WHO). Due to the absence of a unified analysis for Asia in the GBD database, our study combines data from these two WHO regions for a comprehensive assessment. The SEA region comprises Bangladesh, Bhutan, the Democratic People’s Republic of Korea, India, Indonesia, Maldives, Myanmar, Nepal, Sri Lanka, Thailand, and Timor-Leste. Conversely, the WP region encompasses American Samoa, Australia, Brunei Darussalam, Cambodia, China, Cook Islands, Fiji, Guam, Japan, Kiribati, Lao People’s Democratic Republic, Malaysia, Marshall Islands, Micronesia, Mongolia, Nauru, New Zealand, Niue, Northern Mariana Islands, Palau, Papua New Guinea, Philippines, Republic of Korea, Samoa, Singapore, Solomon Islands, Tokelau, Tonga, Tuvalu, Vanuatu, and Vietnam. Our methodology for assessing the burden of NAFLD from the GBD 2019 database has been detailed in a prior study, providing a comprehensive framework for our analysis [13]. The GBD 2019 study assessed data quality on a scale from 0 to 5 to ensure data accuracy. Various statistical methodologies were employed to address data heterogeneity, such as misclassification correction and noise reduction algorithms. The annual prevalence of NAFLD was computed by dividing the number of confirmed cases by the population size. The burden of NAFLD in this age group was assessed using DALYs, which consider Years of Life Lost (YLL) and Years Lived with Disability (YLD). Additional analysis was conducted to explore gender differences. Subsequently, the prevalence and disability-adjusted life years rates were divided in 100,000 populations yielding the terms age-standardized prevalence rates (ASPR) and age-standardized disability-adjusted life years rates (ASDALYs).

Data were collected from various sources, including vital registration systems, verbal autopsies, and surveillance systems, covering the period from 2010 to 2019. These sources standardized cause of death information using ICD 9 and 10 code mapping and merged it into a unified database. This database was used to calculate mortality estimates specific to causes, considering factors such as gender, year, regional location, and age. Processed data are analyzed using standardized tools to generate estimates for each specific parameter of interest, considering factors like age, gender, location, and year. There are three primary standardized tools employed for this purpose: the Cause of Death Ensemble model (CODEm), spatiotemporal Gaussian process regression (ST-GPR), and DisMod-MR. To provide more insight into these general GBD methods, the detail was discussed in the previous GBD publication [10]. In brief, CODEm is a highly structured tool designed for the analysis of cause-of-death data. It utilizes an ensemble of various modeling techniques for rates or causes fractions, with flexible choices of covariates that have demonstrated strong performance through out-of-sample predictive validity testing. DisMod-MR, on the other hand, is a Bayesian meta-regression tool that facilitates the assessment of all available data concerning incidence, prevalence, remission, and mortality for a particular disease. It enforces consistency across various epidemiological parameters. The GBD team divided the burden of mortality from cirrhosis and liver cancer into five categories based on their underlying causes. These categories include viral hepatitis B, viral hepatitis C, alcohol-associated liver disease, NAFLD, and other causes. Lastly, ST-GPR encompasses a set of regression methods that leverage information from different locations and across time for specific metrics of interest, such as risk factor exposure or mortality rates. In addition to these tools, alternative modeling strategies have been developed, particularly for rarer outcomes, in the case of select diseases. The Cause of Death (CoD) database draws from seven different data sources: vital registration (VR), verbal autopsy (VA), sibling history, and surveys/census. In countries where complete vital registration systems are in place, additional data sources are not typically necessary. However, given that less than half of the global population has deaths recorded in a vital registration system, other sources are crucial for comprehensive mortality estimation. Therefore, in countries with incomplete vital registration systems, additional data types are used to supplement vital statistics for causes of death.

The primary source of CoD data is the WHO Mortality Database, which compiles information provided by individual countries. This data is collected from official mortality databases maintained by specific country offices. In cases where possible, CoD is directly coded to the most specific category. However, in some instances, it is coded to broader cause groups based on ICD tabulation lists. These lists include the ICD-9 Basic Tabulation List, ICD-10 Mortality Tabulation, Russia Tabulation, and India Medical Certification of Cause of Death. In countries without VR systems, VA studies are conducted. In VA studies, trained interviewers use standardized questionnaires to gather information from the deceased person’s relatives to determine the cause of death. Additionally, sample registration systems, such as those in Indonesia and India, are increasingly being used. Consequently, VA data exhibit a high degree of heterogeneity because studies employ different instruments, cause lists (ranging from single causes to complete ICD-cause lists), methods for assigning CoD, recall periods, and age groups. Cultural differences may also influence the interpretation of specific questions. When mapping CoD data to the GBD cause, it is important to consider the validity of CoD. VA data may be less accurate for causes that require medical certification, such as diabetes, in comparison to causes like road injuries or homicides [10].

### 2.3. Data and Statistical Analysis

Estimates for incident cases and DALYs were reported with 95% uncertainty intervals (UIs) as 2.5th and 97.5th ranked values across all 1000 draws from a posterior distribution. ASRs were derived using the direct method to the GBD 2019 population estimate [10]. In order to examine changes occurring between 2010 and 2019, we employed a specific formula for analysis: (value in 2019–value in 2010) divided by the value in 2010, calculated for each category. To assess variations in ASRs over this time frame, we utilized Joinpoint regression software, version 4.6.1.0, developed by the Statistical Research and Applications Branch of the National Cancer Institute in Bethesda, MD, USA. This software helped determine the Annual Percentage Change (APC) along with its associated 95% confidence interval (CI). We identified an upward trend when both the yearly rate of change and its lower 95% CI limit were positive. Conversely, a downward trend was observed when both the annual rate of change and its upper boundary were negative.

## 3. Results

### 3.1. Prevalence of Nonalcoholic Fatty Liver Disease in the Elderly in Asia-Pacific

**Figure 1:**
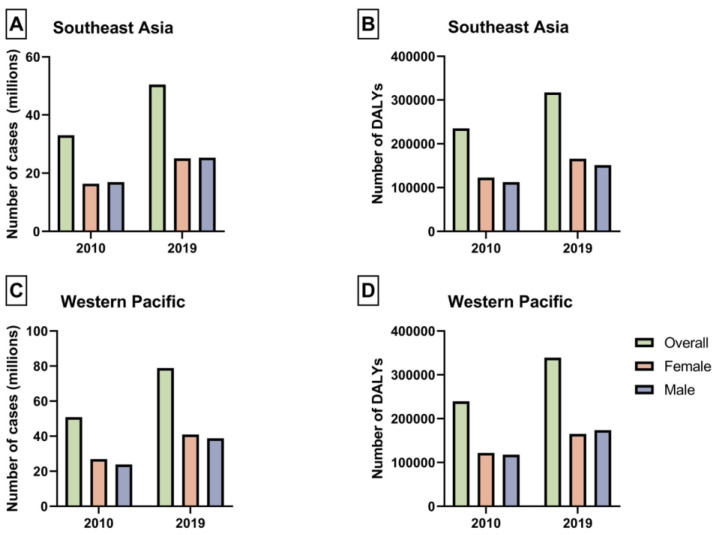
(**A**) Prevalence of nonalcoholic fatty liver disease in patients aged 65–89 in Southeast Asia. (**B**) Frequency of disability-adjusted life years of nonalcoholic fatty liver disease in patients aged 65–89 in Southeast Asia. (**C**) Prevalence of nonalcoholic fatty liver disease in patients aged 65–89 in the Western Pacific region. (**D**) Frequency of disability-adjusted life years of nonalcoholic fatty liver disease in patients aged 65–89 in the Western Pacific region. Figure legends: DALYs: disability-adjusted life years.

**Figure 2:**
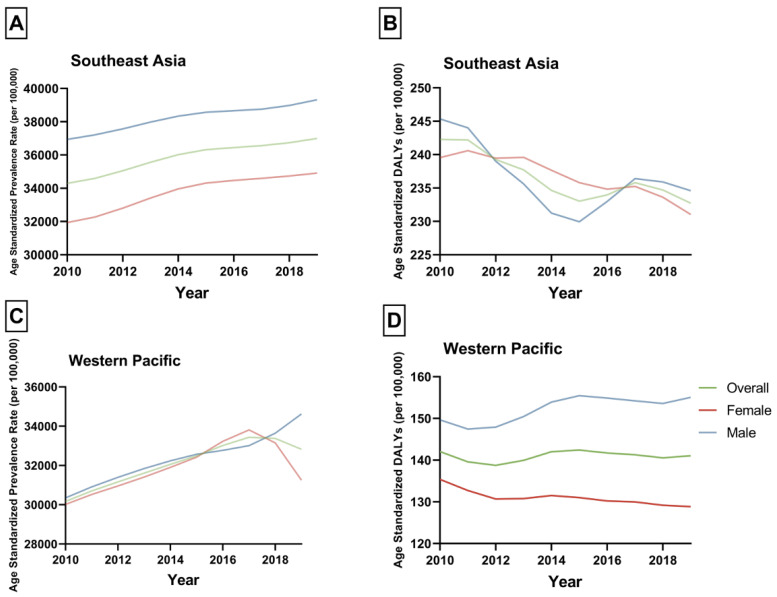
(**A**) Age-standardized prevalence rates per 100,000 of nonalcoholic fatty liver disease in patients aged 65–89 in Southeast Asia. (**B**) Age-standardized disability-adjusted life years per 100,000 of nonalcoholic fatty liver disease in patients aged 65–89 in Southeast Asia. (**C**) Age-standardized prevalence rates per 100,000 of nonalcoholic fatty liver disease in patients aged 65–89 in the Western Pacific region. (**D**) Age-standardized disability-adjusted life years per 100,000 of nonalcoholic fatty liver disease in patients aged 65–89 in the Western Pacific region. Figure legends: DALYs: disability-adjusted life years.

**Figure 3:**
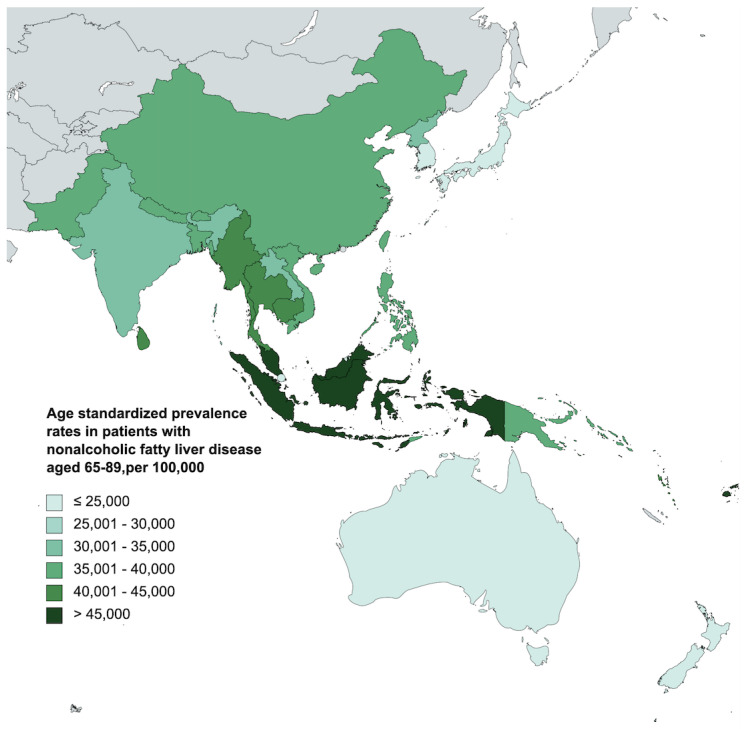
Age-standardized prevalence rates per 100,000 population of nonalcoholic fatty liver disease in patients aged 65–89 years old in Southeast Asia and the Western Pacific region by country.

The frequency of NAFLD prevalence, DALYs, and their rates (ASPRs and ASDALYs) in patients aged 65–89 in Asia are summarized in Table 1 and Table 2. In 2019, the WP had a prevalence of 78.9 million patients with NAFLD in the elderly (Figure 1C). SEA exhibited a prevalence of 50.41 million (Figure 1A). The ASPR in WP and SEA were 32,821.78 (95% UI 27,603.74 to 38,673.58) and 36,995.37 (95% UI 30,793.47 to 44,330.06) per 100,000, respectively (Figure 2A,C). From 2010 to 2019, the fastest-growing prevalence rate of NAFLD in the elderly was observed in the WP region (APC: +0.95%, 95% CI 0.87 to 1.03%) (Table 1)with higher growth in males (APC: 1.3%, 95% CI 1.12 to 1.48%) than in females (APC: 0.59%, 95% CI 0.35 to 0.83%). In contrast, the SEA region exhibited a higher degree of increase in females (APC: 1.01%, 95% CI 0.9 to 1.1%) than males (Table 1). Stratified by country, the most pronounced increase was seen in India (APC: 1.21%, 95% CI 1.09 to 1.34%) (Supplementary Material Table S1). However, the highest ASPR in 2019 was noted in Malaysia, with 48,314.12 (95% UI: 41,420.31 to 55,450.66) per 100,000. A graphical depiction of national variations in ASPR can be found in Figure 3.

### 3.2. Disability-Adjusted Life Years of Nonalcoholic Fatty Liver Disease in the Elderly in Asia-Pacific

Regarding disability, in 2019, the WP had 339,102 DALYs (Figure 1D). SEA, on the other hand, had 317,071 DALYs (Figure 1B). The ASDALYs in WP and SEA were 141.06 (95% UI 107.79 to 181.26) and 232.71 (95% UI 164.97 to 317.25) per 100,000, respectively (Figure 2B,D). Between 2010 and 2019, SEA demonstrated a decline in disability (APC: −0.41%, 95% CI −0.66 to −0.17) (Table 2) with higher decrease in males’ ASDALYs (APC: −0.48%, 95% CI −0.86 to −0.11%) than females (APC: −0.42%, 95% CI −0.53 to −0.31%). On the contrary, the WP region displayed a stable trend in ASDALYs. However, the overall pattern concealed the gender variation, with a noticeable rise in ASDALYs observed among males (APC: 0.55%, 95% CI 0.24 to 0.87%), whereas a decreasing trend in ASDALYs was discernible among females in the same region (APC: −0.5%, 95% CI −0.73 to −0.26%). When analyzed nationally, the most notable increase was observed in Timor-Leste (APC: 2.11%, 95% CI 1.41 to 2.83%) (Supplementary Material Table S2). However, the highest ASDALYs in 2019 were observed in Cambodia, with 795.79 (95% UI: 494.65 to 1162.46) per 100,000.

## 4. Discussion

To our knowledge, this study represents the first comprehensive examination of NAFLD burden among elderly populations in the Asia-Pacific region spanning a decade (2010 to 2019). In 2019, the Asia-Pacific region witnessed an alarming increase in NAFLD cases among the elderly, exceeding 120 million cases. This increase was observed across both genders, with a more pronounced increase in males in the Western Pacific region, and females in the Southeast Asia region. In contrast to the ASR of prevalence, the ASR of DALYs associated with NAFLD in the elderly decreased in Southeast Asia but remained relatively stable in the Western Pacific region. Notably, there was significant heterogeneity in the burden of NAFLD in the elderly among various countries within the Asia-Pacific region. These findings shed light on the increasing impact of NAFLD among the elderly population, underlining the need for region-specific strategies and interventions to address this growing health concern.

Aligned with previous studies, our study found that the burden of NAFLD in the elderly was higher. This could be attributable to the higher burden of comorbidities in this group of population along with aging which includes increased inflammation (termed inflame-aging) and dysfunction of mitochondria [14,15]. In addition, elderly individuals are disproportionately impacted by NAFLD and its associated complications, including the progression of hepatic and extrahepatic complications [6,14]. However, when interpreting the burden of NAFLD in the elderly in comparison to the younger population, caution is advised. This is due to the fact that individuals with NAFLD tend to be at an elevated risk of developing cardiometabolic complications, which could potentially introduce a selection bias into this elderly group [16,17]. Furthermore, it is important to note that there are currently no specific guidelines offering recommendations for diagnostic procedures to be followed in elderly individuals with NAFLD [4].

In terms of gender differences, our study found that the NAFLD burden was higher in elderly males, consistent with a prior study using the same global database [2]. However, several prior investigations have suggested that in elderly populations, the burden of NAFLD in females either equals or exceeds that in males, potentially due to the protective effects of estrogen waning after menopause [18,19]. To comprehensively comprehend these gender disparities, further extensive research is essential. It is crucial to explore these distinctions as females and males exhibit metabolic variances, with unique regulatory factors influencing sex-specific metabolic outcomes. This understanding can offer valuable insights into tailored approaches for the prevention and management of NAFLD in elderly populations [20,21]. From a physiological perspective, the liver stands as the second most sexually distinct organ in the human body [22]. These distinctions encompass various factors, including but not limited to the metabolism of fatty acids and mitochondrial processes [23]. These differences can contribute to variations in the burden of NAFLD between the two sexes. Nonetheless, even though our study observed a lower NAFLD burden in elderly females, it is crucial not to overlook the burden in this demographic. Elderly females often experience a heightened burden of chronic diseases, disabilities, and comorbidities, with this burden being most pronounced among socioeconomically disadvantaged and minority women [24,25]. Therefore, policymakers should also take gender disparities into account when tailoring strategies for managing this condition in the elderly population of the Asia-Pacific region, which comprises a diverse range of populations.

The extensive geographical expanse in Asia could explain the heterogeneity in NAFLD burden, and there is substantial heterogeneity in terms of ethnicities, lifestyles, economic conditions, and disease patterns [26]. Despite national differences, our study revealed consistent findings with implications for younger cohorts; NAFLD affects the elderly in all demographics across the Asia-Pacific region regardless of socioeconomic status [27]. This observed pattern corresponds with the rising occurrence of obesity and diabetes throughout the continent, crucial contributory factors for NAFLD [28,29]. Similar to other metabolic disorders, the prevalence of NAFLD is higher in the elderly than in their younger counterparts [30]. This is a matter of concern since the liver-related burden of metabolic dysfunction-associated steatohepatitis is increasing. It is also vital to view NAFLD not just as a standalone liver disease but as a holistic disorder closely tied to significant cardiometabolic complications [31,32]. In relation to the Asian demographics, which are the majority of the population in the Asia-Pacific region, they have a heightened propensity to encounter these cardiometabolic risks and type 2 diabetes at a relatively lower body mass index compared to their Western counterparts [7]. Furthermore, several studies have shown that Asians have a higher proportion of lean NAFLD cases, in which individuals with NAFLD are not overweight [33]. These lean individuals with NAFLD are at a greater risk of developing atherosclerotic complications and experiencing higher mortality rates [34,35,36]. Collectively, the significant impact NAFLD exerts on the aging population in the Asia-Pacific and the consequential strain it exerts on the healthcare system is a matter that cannot be overemphasized.

Regarding disability, the ASDALYs showed a decline in SEA and remained stable in the WP region, which could be attributed to improvements in the healthcare system and public policy. However, it is important to note that despite these improvements, the burden of NAFLD in the elderly remains high, and the observed decline in burden may be influenced by underreporting in the GBD database [10]. In nations with inadequate data sources, the GBD predominantly relies on modeling methods, predictive factors, historical patterns, or trends inferred from surrounding countries. This creates a degree of uncertainty when calculating the actual burden of NAFLD [10,37]. Given the unique presentation of NAFLD in older populations, particularly among Asian demographics, it is crucial to classify and understand this specific patient group accurately. Such classification would enable better prediction of disease outcomes and customization of suitable treatment strategies. Regrettably, older individuals, especially Asian, are frequently underrepresented in clinical trials, presenting an additional challenge in determining whether a treatment’s potential benefits outweigh its associated risks [38]. Nonetheless, the existing guidelines do not provide explicit recommendations regarding the diagnostic approach or distinctive features in this demographic, such as comorbidities, that should be employed when addressing NAFLD in the elderly population [39]. It will require time to develop a solid understanding, leading to practical guidelines for this specific group. For these reasons, the primary preventative measures concerning the risk factors, including adherence to a healthy lifestyle, continue to be the optimal approach to prevent and mitigate the detrimental effect of NAFLD impact on individual health. Moreover, personalized medicine can play a significant role in managing NAFLD including metabolic syndrome [40]. Given that NAFLD affects more than a quarter of the population, it is impractical to screen everyone [41]. A recent study demonstrated that specific genetic variants, such as patatin-like phospholipase domain-containing 3 (PNPLA3), or biomarkers, can possibly be used to predict the progression of NAFLD [42,43,44]. In a retrospective study by Chen et al., those with an intermediate risk (FIB4 score between 1.3 and 2.67), combined with the PNPLA3 rs738409-GG genotype and diabetes, displayed a cirrhosis risk similar to high-risk individuals (FIB4 score >2.67). This highlights the potential of the PNPLA3 rs738409-GG genotype and diabetes presence in stratifying cirrhosis risk in NAFLD patients [45]. However, several challenges still need to be addressed, including the necessity to validate these findings across diverse populations. In addition, given the complexity of NAFLD, multiple computational models have been created mimicking the different functions of the liver, the most complex organ in terms of metabolism [46,47]. With the advent of novel computation models, the simulation could predict the outcome of the patient and ensure treatment and the ability to predict the prognosis in the future [48]. Yet, further research is essential, especially in the context of elderly individuals with NAFLD, to understand the genetic factors and potential outcomes of NAFLD in various subgroups of the population.

While this study provides a thorough evaluation of NAFLD in the elderly population within the Asia-Pacific region, it is essential to acknowledge several inherent limitations. Firstly, the accuracy of the GBD data is contingent upon the quality and extent of vital registration systems in each country, and this limitation is especially pronounced in nations with limited available data [10]. The GBD estimates heavily rely on modeling techniques, predictive variables, historical trends, or extrapolations from nearby nations. Secondly, employing ICD codes to estimate NAFLD prevalence in the elderly population may lead to potential underreporting and misclassification. Caution is advised when interpreting the findings, and future investigations could benefit from combining multiple data sources to gain a more comprehensive understanding of the NAFLD burden in this demographic. Thirdly, it is important to consider the methodology of GBD, which initially computes the overall liver-related burden, including the decreasing burden of viral hepatitis. As a result, the interpretation of DALYs attributed to NAFLD, particularly when there is a negative trend in mortality or disability, should be approached with caution [10,37]. Therefore, it is crucial to be mindful of this interpretation suggesting a decrease in disability and consider the broader context.

## 5. Conclusions

The prevalence of NAFLD amongst the elderly has witnessed a significant surge from 2010 to 2019 in the Asia-Pacific region, with a more pronounced burden in males. This rapid surge is a major concern, framing NAFLD as a rising public health challenge at both the national and regional levels, especially among at-risk groups like the elderly.

## Figures and Tables

**Table 1:** Age-standardized prevalence rates of nonalcoholic fatty liver disease in patients aged 65–89 years in 2010 and 2019 in Western Pacific and Southeast Asia, stratified by sex.

	2010 Prevalence (95% UI)	2010 ASPR, per 100,000 (95% UI)	2019 Prevalence (95% UI)	2019 ASPR, per 100,000 (95% UI)	APC (95% CI)	*p*
Western Pacific						
Overall	50,872,617.88 (42,425,025.12 to 60,674,143.65)	30,171.62 (25,161.51 to 35,984.73)	78,901,129.54 (66,357,352.99 to 92,968,439.78)	32,821.78 (27,603.74 to 38,673.58)	0.95 (0.87 to 1.03)	<0.001
Female	26,989,633.59 (22,468,540.21 to 32,562,717.23)	30,017.44 (24,989.15 to 36,215.73)	40,095,703.93 (33,317,904.88 to 48,768,977.91)	31,247.77 (25,965.63 to 38,007.11)	0.59 (0.35 to 0.83)	<0.001
Male	23,882,984.29 (19,905,539.39 to 28,371,702.26)	30,347.79 (25,293.7 to 36,051.54)	38,805,425.61 (32,892,635.6 to 45,127,982.77)	34,623.83 (29,348.19 to 40,265.09)	1.3 (1.12 to 1.48)	<0.001
Southeast Asia						
Overall	33,306,919.91 (27,504,062.2 to 40,512,069.6)	34,295.03 (28,320.02 to 41,713.93)	50,406,719.89 (41,956,546.18 to 60,400,342.45)	36,995.37 (30,793.47 to 44,330.06)	0.87 (0.78 to 0.95)	<0.001
Female	16,388,617.1 (13,407,026.03 to 20,105,405.24)	31,935.36 (26,125.34 to 39,178.01)	25,081,401.08 (20,780,159.13 to 30,536,791.54)	34,912.59 (28,925.38 to 42,506.33)	1.01 (0.9 to 1.11)	<0.001
Male	16,918,302.81 (14,079,388.13 to 20,414,306.75)	36,938.96 (30,740.55 to 44,572.04)	25,325,318.8 (21,207,566.73 to 30,006,548.71)	39,318.41 (32,925.46 to 46,586.17)	0.68 (0.57 to 0.79)	<0.001

Abbreviations: ASPR—age-standardized prevalence rates; APC—annual percentage change; CI—confidence interval; UI—uncertainty interval.

**Table 2:** Age-specific disability-adjusted life years of nonalcoholic fatty liver disease in patients aged 65–89 years in 2010 and 2019 in Western Pacific and Southeast Asia, stratified by sex.

	2010 DALYs (95% UI)	2010 ASDALYs, per 100,000 (95% UI)	2019 DALYs (95% UI)	2019 ASDALYs, per 100,000 (95% UI)	APC (95% CI)	*p*
Western Pacific						
Overall	239,498.28 (181,736.79 to 306,983.96)	142.04 (107.78 to 182.07)	339,101.73 (259,107.34 to 435,733.54)	141.06 (107.79 to 181.26)	0.0 (−0.15 to 0.3)	0.485
Female	121,739.38 (93,158.61 to 154,664.67)	135.4 (103.61 to 172.02)	165,325.16 (124,489.45 to 214,151.49)	128.84 (97.02 to 166.89)	−0.5 (−0.73 to −0.26)	<0.001
Male	117,758.9 (88,314.9 to 153,076.43)	149.63 (112.22 to 194.51)	173,776.58 (130,264.88 to 228,502.19)	155.05 (116.23 to 203.88)	0.55 (0.24 to 0.87)	0.003
Southeast Asia						
Overall	235,306.26 (167,996.37 to 319,919.62)	242.29 (172.98 to 329.41)	317,070.93 (224,777.19 to 432,256.57)	232.71 (164.97 to 317.25)	−0.41 (−0.66 to −0.17)	0.001
Female	122,934.42 (86,730.17 to 167,482.56)	239.55 (169.01 to 326.36)	165,970.08 (117,185.61 to 225,328.43)	231.03 (163.12 to 313.65)	−0.42 (−0.53 to −0.31)	<0.001
Male	112,371.83 (78,463.65 to 153,263.92)	245.35 (171.32 to 334.63)	151,100.85 (105,089.3 to 210,042.2)	234.59 (163.15 to 326.1)	−0.48 (−0.86 to −0.11)	0.012

Abbreviations: ASDALYs—age-standardized disability rates; APC—annual percentage change; CI—confidence interval; DALYs—disability-adjusted life years; UI—uncertainty interval.

## Data Availability

The Global Burden of Disease study in 2019 offers comprehensive data on the burden of diseases and risk factors across 204 countries and territories. Access to these data is provided by the GlobalHealth Data Exchange query tool (http://ghdx.healthdata.org/gbd-results-tool, accessed on 10 March 2023), which the Institute for Health Metrics and Evaluation maintains.

## Supplementary Materials

The following supporting information can be downloaded at: https://www.mdpi.com/article/10.3390/jcm12206456/s1, Table S1: Age-standardized prevalence rates of nonalcoholic fatty liver disease in patients aged 65–89 years in 2010 and 2019, stratified by countries in Asia-Pacific Region; Table S2: Age-standardized disability-adjusted life years of nonalcoholic fatty liver disease in patients aged 65–89 years in 2010 and 2019, stratified by countries in Asia-Pacific Region.
