# Supplementary material for: The Surreptitious Burden of Nonalcoholic Fatty Liver Disease in the Elderly in the Asia-Pacific Region: An Insight from the Global Burden of Disease Study 2019
Source: J Clin Med. 2023 Oct 11;12(20):6456. doi: 10.3390/jcm12206456 (PMC10607093; doi:10.3390/jcm12206456)
Supplement: Supplementary file 1 [file jcm-12-06456-s001.zip › jcm-2597345-supplementary.pdf]

Supplementary Material 1 Age-standardized prevalence rates of nonalcoholic fatty liver disease in patients aged 65-89 years in 2010 and 2019, stratified by countries in the Asia Pacific Region

Countries	2010 ASPR per 100,000 (95% UI)	2019 ASPR per 100,000 (95% UI)	APC (95% CI)	*p*
American Samoa	47171.43 (40717.96 to 53629.2)	47106.55 (40579.11 to 53526.79)	-0.01 (-0.03 to 0)	0.151
Australia	20845.75 (17632.28 to 24143.29)	21478.11 (18161.41 to 25013.14)	0.31 (0.29 to 0.34)	**<0.001**
Bangladesh	34615.85 (28375.41 to 43128)	36361.18 (29907.85 to 44265.55)	0.53 (0.5 to 0.57)	**<0.001**
Bhutan	36795.3 (29992.74 to 45328.27)	38796.75 (32307.13 to 46330.71)	0.59 (0.56 to 0.62)	**<0.001**
Brunei Darussalam	18767.3 (15271.75 to 22933.08)	19017.28 (15453.61 to 23417.47)	0.13 (0.1 to 0.15)	**<0.001**
Cambodia	39495.36 (32928.96 to 46860.05)	40373.92 (33633.18 to 47833.37)	0.24 (0.23 to 0.25)	**<0.001**
China	33656.54 (28011.26 to 40325.2)	36249.83 (30394.6 to 42836.55)	0.84 (0.75 to 0.93)	**<0.001**
Cook Islands	45371.79 (38907.91 to 51917.79)	46343.59 (40118.25 to 52642.77)	0.23 (0.21 to 0.25)	**<0.001**
Democratic People's Republic of Korea	32044.09 (26151.64 to 39797.85)	33531.46 (27611.91 to 40516.11)	0.52 (0.5 to 0.54)	**<0.001**
Fiji	44814.35 (37563.75 to 52041.41)	46036.96 (39402.96 to 52919.16)	0.31 (0.28 to 0.33)	**<0.001**
Guam	40769.55 (34969.42 to 46526.74)	41574.63 (35586.56 to 47388.07)	0.22 (0.21 to 0.22)	**<0.001**
India	31363.9 (25576.03 to 38998.1)	34836.55 (28794.45 to 42218.24)	1.21 (1.09 to 1.34)	**<0.001**
Indonesia	45066.75 (38131.09 to 52381.84)	46139.35 (39222.91 to 53348.37)	0.26 (0.25 to 0.27)	**<0.001**
Japan	15838.67 (13166.58 to 19045.66)	17231.42 (14366.11 to 20530.71)	0.98 (0.92 to 1.03)	**<0.001**
Kiribati	42830.95 (36122.19 to 49941.71)	42861.33 (36032.26 to 49928.46)	0.01 (-0.01 to 0.03)	0.235
Lao People's Democratic Republic	33350.97 (27707.62 to 39720.91)	33959.85 (28307.22 to 40357.39)	0.21 (0.17 to 0.25)	**<0.001**
Malaysia	46921.38 (39979.7 to 54164.86)	48314.12 (41420.31 to 55450.66)	0.32 (0.3 to 0.35)	**<0.001**
Maldives	41568.5 (35113.66 to 48545.87)	42698.81 (36550.72 to 49201.38)	0.28 (0.23 to 0.33)	**<0.001**
Marshall Islands	38373.55 (31873.77 to 45235.14)	39259.79 (32447.31 to 46525.02)	0.27 (0.22 to 0.32)	**<0.001**
Mauritius	35231.22 (29867.03 to 40892.82)	36006.88 (30661.96 to 41326.27)	0.24 (0.23 to 0.25)	**<0.001**
Micronesia	42440.78 (36376.76 to 48803.01)	41892.8 (35399.22 to 48516.48)	-0.15 (-0.17 to -0.13)	**<0.001**
Myanmar	41894.53 (35191.41 to 49381.02)	42797.03 (36104.2 to 50512.18)	0.23 (0.22 to 0.24)	**<0.001**
Nauru	39995.13 (33583.65 to 47060.76)	42851.34 (35846.16 to 50114.01)	0.8 (0.77 to 0.84)	**<0.001**
Nepal	33460.13 (27351.62 to 41534.49)	36531.3 (29963.73 to 44474.21)	0.98 (0.94 to 1.02)	**<0.001**
New Zealand	18812.49 (15961.77 to 21786.75)	19441.21 (16439.45 to 22519.34)	0.36 (0.35 to 0.38)	**<0.001**

Niue	44069.85 (38100.8 to 50378.07)	44930.06 (38673.6 to 51097.61)	0.22 (0.21 to 0.24)	**<0.001**
Northern Mariana Islands	44609.22 (38310.62 to 51010.9)	44181.1 (37453.72 to 50792.61)	-0.14 (-0.27 to 0)	**0.044**
Pakistan	38497.96 (31611.97 to 46531.5)	39926.83 (33130.67 to 47952.77)	0.41 (0.39 to 0.42)	**<0.001**
Palau	44497.77 (38077.38 to 50901.66)	44989.68 (38383.26 to 51773.8)	0.13 (0.1 to 0.15)	**<0.001**
Papua New Guinea	36744.22 (30324.14 to 44081.93)	37207.02 (30427.38 to 44666.33)	0.15 (0.12 to 0.18)	**<0.001**
Philippines	35020.05 (29333.2 to 41161.5)	35504.24 (29944.62 to 41523.99)	0.15 (0.15 to 0.16)	**<0.001**
Republic of Korea	18913.28 (16480.33 to 21364.21)	18473.18 (15366.15 to 22183)	-0.52 (-1.52 to 0.49)	0.313
Samoa	42954.78 (36864.08 to 49561.66)	43000.92 (37025.76 to 49429.66)	0.02 (0.01 to 0.04)	**0.014**
Seychelles	44442.82 (38199.43 to 51256.74)	45584.07 (39273.4 to 52112.71)	0.28 (0.25 to 0.3)	**<0.001**
Singapore	20975.89 (17271.11 to 25677.52)	21630.5 (17877.46 to 26139.32)	0.32 (0.27 to 0.37)	**<0.001**
Solomon Islands	39351.16 (32305.33 to 46695.99)	39446.95 (32561.07 to 46571.7)	0.04 (0 to 0.09)	0.052
Sri Lanka	39715.64 (33072.62 to 46657.79)	40724.44 (34023.11 to 47779.54)	0.28 (0.26 to 0.31)	**<0.001**
Taiwan	36879.75 (31432.79 to 42999.21)	38603.28 (32612.04 to 45030.16)	0.49 (0.45 to 0.53)	**<0.001**
Thailand	41240.46 (34817.54 to 47699.37)	42615.52 (36512.38 to 48751.9)	0.35 (0.3 to 0.4)	**<0.001**
Timor-Leste	37334.97 (30524.14 to 45260.88)	37905.1 (31205.63 to 45457.01)	0.22 (0.17 to 0.26)	**<0.001**
Tokelau	40999.21 (34963.04 to 47323.18)	42409.06 (36110.53 to 48822.51)	0.42 (0.38 to 0.47)	**<0.001**
Tonga	43714.06 (37450.83 to 50113.64)	43929.42 (37731.21 to 50171.42)	0.05 (0.03 to 0.07)	**<0.001**
Tuvalu	40100.1 (33840.86 to 46882.77)	40848.09 (34196.7 to 47608.97)	0.22 (0.19 to 0.24)	**<0.001**
Vanuatu	41230.15 (34432.95 to 48145.06)	41896.79 (35185.42 to 48919.42)	0.18 (0.16 to 0.19)	**<0.001**
Viet Nam	35675.12 (29593.09 to 42355.75)	36449.16 (30539.6 to 43021.33)	0.24 (0.23 to 0.26)	**<0.001**

APC, annual percentage change; ASIR, Age-standardized incidence rate ; 95% CI, 95% confidence interval; 95% UI, 95% uncertainty interval

Supplementary Material 2 Age-standardized disability-adjusted life years of nonalcoholic fatty liver disease in patients aged 65-89 years in 2010 and 2019, stratified by countries in the Asia Pacific Region

**Countries**	**2010 ASDALYs per 100,000 (95% UI)**	**2019 ASDALYs per 100,000 (95% UI)**	**APC (95% CI)**	***p***
American Samoa	204.24 (145.7 to 283.37)	201.25 (141.3 to 274.95)	-0.17 (-0.29 to -0.05)	**0.006**
Australia	124.33 (93.21 to 164.26)	130.32 (96.08 to 172.5)	0.4 (-0.2 to 1.01)	0.19
Bangladesh	131.7 (85.25 to 192.41)	129.74 (82.63 to 196.13)	0.12 (-1.29 to 1.54)	0.871
Bhutan	218.51 (133.13 to 379.74)	218.9 (132.33 to 376.68)	0.07 (-0.08 to 0.22)	0.366
Brunei Darussalam	139.71 (94.28 to 200.85)	124.03 (84.91 to 176.21)	-1.14 (-1.39 to -0.88)	**<0.001**
Cambodia	682.49 (435.02 to 987.26)	795.79 (494.65 to 1162.46)	1.79 (1.43 to 2.15)	**<0.001**
China	132.15 (98.97 to 171.94)	133.54 (101.02 to 174.18)	0.44 (0.04 to 0.85)	**0.036**
Cook Islands	223.25 (153.71 to 310.77)	225.31 (156.6 to 320.61)	0.08 (-0.07 to 0.24)	0.247
Democratic People's Republic of Korea	191.19 (120.95 to 273.62)	201.8 (128.84 to 288.51)	0.77 (0.4 to 1.15)	**<0.001**
Fiji	172.93 (122.63 to 238.97)	182.22 (122.77 to 258.84)	0.54 (0.48 to 0.6)	**<0.001**
Guam	164.59 (113.86 to 229.65)	177.62 (121.37 to 250.15)	0.92 (0.55 to 1.29)	**<0.001**
India	159.11 (112.77 to 217.4)	155.34 (107.93 to 215.8)	-0.25 (-0.68 to 0.18)	0.247
Indonesia	657.21 (432.04 to 933.08)	617.35 (406.33 to 880.16)	-0.75 (-1.23 to -0.26)	**0.003**
Japan	102.8 (81.43 to 124.91)	86.73 (67.65 to 106.24)	-2.04 (-2.61 to -1.46)	**<0.001**
Kiribati	430.74 (279.8 to 639.07)	400.89 (254.26 to 602.73)	-0.79 (-0.93 to -0.64)	**<0.001**
Lao People's Democratic Republic	278.37 (172.12 to 426.93)	307.12 (197.35 to 471.01)	1.14 (0.93 to 1.35)	**<0.001**
Malaysia	289.81 (207.67 to 384.68)	289.17 (189.75 to 410.57)	-0.23 (-0.57 to 0.11)	0.177
Maldives	195.9 (141.88 to 268.68)	202.62 (137.85 to 282.55)	0.37 (0.28 to 0.47)	**<0.001**
Marshall Islands	291.53 (171.13 to 445.58)	295.21 (174.67 to 475.97)	0.14 (0.08 to 0.2)	**<0.001**
Mauritius	178.78 (122.65 to 250.68)	158.67 (104.81 to 224.01)	-1.42 (-2.19 to -0.64)	**<0.001**
Micronesia	320.83 (181.99 to 514.21)	323.51 (184.01 to 515.67)	0.05 (-0.16 to 0.26)	0.618
Myanmar	232.43 (151.56 to 336.56)	269.56 (180.94 to 381.23)	1.79 (1.26 to 2.33)	**<0.001**
Nauru	330.16 (197.87 to 510.6)	315.41 (181.34 to 503.33)	-0.5 (-0.6 to -0.41)	**<0.001**
Nepal	207.39 (126.59 to 339.84)	247.78 (147.74 to 400.26)	2.11 (1.45 to 2.77)	**<0.001**

New Zealand	109.79 (85.99 to 140.02)	114.55 (89.76 to 143.8)	0.33 (-0.5 to 1.16)	0.436
Niue	283.88 (187.43 to 414.58)	271.06 (177.68 to 387.12)	-0.52 (-0.55 to -0.49)	**<0.001**
Northern Mariana Islands	290.75 (200.12 to 404.86)	274.81 (186.5 to 378.09)	-0.62 (-0.94 to -0.31)	**<0.001**
Pakistan	206.24 (111.42 to 340.25)	181.77 (104.46 to 299.49)	-1.47 (-1.57 to -1.37)	**<0.001**
Palau	245.32 (151.3 to 374.24)	233.09 (145.12 to 357.68)	-0.53 (-0.62 to -0.45)	**<0.001**
Papua New Guinea	71.91 (44.12 to 110.33)	75.23 (45.68 to 116.93)	0.46 (0.08 to 0.85)	**0.017**
Philippines	231.34 (165.43 to 308.1)	244.71 (175.46 to 333.88)	0.69 (0.48 to 0.9)	**<0.001**
Republic of Korea	177.51 (123.94 to 250.33)	159.14 (104.89 to 229.95)	-1.22 (-1.35 to -1.08)	**<0.001**
Samoa	208.94 (137.77 to 306.74)	205.8 (137.45 to 298.53)	-0.17 (-0.35 to 0.01)	0.057
Seychelles	357.07 (244.35 to 504.57)	356.47 (243.08 to 494.64)	0.06 (-0.18 to 0.31)	0.618
Singapore	91.36 (63.03 to 131.9)	79 (52.29 to 116.06)	-1.74 (-2.26 to -1.21)	**<0.001**
Solomon Islands	225.47 (145.85 to 332.89)	225.85 (143.85 to 328.12)	-0.18 (-0.56 to 0.2)	0.357
Sri Lanka	200.13 (133.72 to 282.08)	215.71 (133.71 to 321.4)	0.98 (0.55 to 1.41)	**<0.001**
Taiwan	318.98 (225.63 to 430.3)	266.07 (175.52 to 389.3)	-2.12 (-2.67 to -1.57)	**<0.001**
Thailand	506.96 (360.71 to 686.9)	507.84 (345.41 to 728.32)	-0.03 (-0.19 to 0.13)	0.679
Timor-Leste	270.71 (156.59 to 454.52)	323.07 (193.79 to 533.24)	2.11 (1.41 to 2.83)	**<0.001**
Tokelau	222.3 (149.35 to 323.94)	210.37 (139.86 to 313.25)	-0.65 (-0.87 to -0.43)	**<0.001**
Tonga	491.36 (340.96 to 682.5)	491.78 (342.02 to 683.7)	-0.04 (-0.09 to 0.02)	0.162
Tuvalu	251.15 (154.23 to 379.39)	256.36 (160.35 to 385.78)	0.27 (0.16 to 0.37)	**<0.001**
Vanuatu	282.69 (167.06 to 440.79)	272.05 (157.38 to 411.11)	-0.52 (-0.73 to -0.31)	**<0.001**
Viet Nam	267.05 (177.59 to 387.7)	292.17 (180.53 to 429.14)	0.88 (0.31 to 1.45)	**0.002**

APC, annual percentage change; ASDALYs, Age-standardized disability-adjusted life years DALYs, disability-adjusted life years; 95% CI, 95% confidence interval; 95% UI, 95% uncertainty interval
